# Prepulse inhibition of auditory change-related cortical responses

**DOI:** 10.1186/1471-2202-13-135

**Published:** 2012-10-31

**Authors:** Koji Inui, Aki Tsuruhara, Minori Kodaira, Eishi Motomura, Hisashi Tanii, Makoto Nishihara, Sumru Keceli, Ryusuke Kakigi

**Affiliations:** 1 Department of Integrative Physiology, National Institute for Physiological Sciences, Okazaki, 444-8585, Japan; 2 Department of Psychiatry, Mie University Graduate School of Medicine, Tsu, 514-8507, Japan; 3 Multidisciplinary Pain Center, Aichi Medical University, Aichi, 480-1195, Japan

## Abstract

**Background:**

Prepulse inhibition (PPI) of the startle response is an important tool to investigate the biology of schizophrenia. PPI is usually observed by use of a startle reflex such as blinking following an intense sound. A similar phenomenon has not been reported for cortical responses.

**Results:**

In 12 healthy subjects, change-related cortical activity in response to an abrupt increase of sound pressure by 5 dB above the background of 65 dB SPL (test stimulus) was measured using magnetoencephalography. The test stimulus evoked a clear cortical response peaking at around 130 ms (Change-N1m). In Experiment 1, effects of the intensity of a prepulse (0.5 ~ 5 dB) on the test response were examined using a paired stimulation paradigm. In Experiment 2, effects of the interval between the prepulse and test stimulus were examined using interstimulus intervals (ISIs) of 50 ~ 350 ms. When the test stimulus was preceded by the prepulse, the Change-N1m was more strongly inhibited by a stronger prepulse (Experiment 1) and a shorter ISI prepulse (Experiment 2). In addition, the amplitude of the test Change-N1m correlated positively with both the amplitude of the prepulse-evoked response and the degree of inhibition, suggesting that subjects who are more sensitive to the auditory change are more strongly inhibited by the prepulse.

**Conclusions:**

Since Change-N1m is easy to measure and control, it would be a valuable tool to investigate mechanisms of sensory gating or the biology of certain mental diseases such as schizophrenia.

## Background

Prepulse inhibition (PPI) is a phenomenon whereby a weak leading stimulus, or prepulse, inhibits startling reflexes evoked by a subsequent intense abrupt stimulus [1]. PPI is commonly considered a preattentional inhibitory process called sensorimotor gating, by which sensory information is screened so that an individual can focus on the most salient aspects of the sensory environment [2,3]. PPI is useful for investigating mechanisms of sensory filtering because it is common to all mammals, which enables findings to be compared between humans and animals [4]. Clinically, PPI has been repeatedly shown to be impaired in patients with schizophrenia [4,5] and unaffected first-degree relatives of probands [6,7]. Therefore, PPI is an important tool to investigate the biology of schizophrenia. Usually, an intense sound is used to elicit auditory startle responses. The blink reflex is measured in humans by using electromyography and whole-body flinching is measured in rodents by using stabilimeter chambers.

Change-related cortical responses are a sensory-evoked cortical activation specific to a change of stimulus, and recorded very clearly with electroencephalography (EEG) or magnetoencephalography (MEG). The change-related response is elicited without any tasks and without the subject’s attention by any sensory changes including the onset and offset of a stimulus in the auditory [8-12], somatosensory [13,14] and visual [15-17] systems. In addition to sensory-specific areas, multi-modal cortical regions that specifically respond to sensory changes are shown in functional magnetic resonance imaging (fMRI) and MEG studies [18,19]. Since the automatic change-detecting system is thought to play an important role in the quick detection of changes in the sensory environment and therefore in survival, it can be considered a subtype of defense reactions like the startle reflex is [20]. As change-related responses are based on the comparison of a new sensory event with the preceding status, sensory memory and a comparison process are involved in generating them. In fact, the latency and magnitude of change-related responses depends on the duration [9-11,14,17,19,21,22] and intensity [9,12] of the preceding stimulus, in addition to the magnitude of the change itself [9,23].

Based on the similarity between the startle reflex and change-related cortical response with respect to both physiological significance and experimental behavior, we hypothesized that the change-related cortical response is inhibited by a preceding weak stimulus in a similar manner to PPI. Given the defensive and attentional role of the change-related response, the processing of a leading weak change should be protected from being drowned out by a subsequent greater change. To test this, we recorded auditory change-related cortical responses using MEG, and examined effects of a preceding weak change (prepulse) in the present study.

## Methods

The study was approved in advance by the Ethics Committee of the National Institute for Physiological Sciences, Okazaki, Japan, and written consent was obtained from all the subjects. The experiment was performed on twelve (four females and eight males) healthy volunteers, aged 25–53 (38.5 ± 7.4) years. They were asked to refrain from alcohol, caffeine and smoking for at least 12 hours prior to the experiment. There were two smokers. All subjects were without a history of mental or neurological disorders, or substance abuse in the last two years. They were free of medication at testing. All subjects underwent a psychiatric assessment using the Mini-International Neuropsychiatric Interview [24], and had a hearing threshold lower than 30 dB at 1000 Hz as assessed by an audiometer (AA-71, Rion, Tokyo, Japan).

### Auditory stimuli

**Figure 1:**
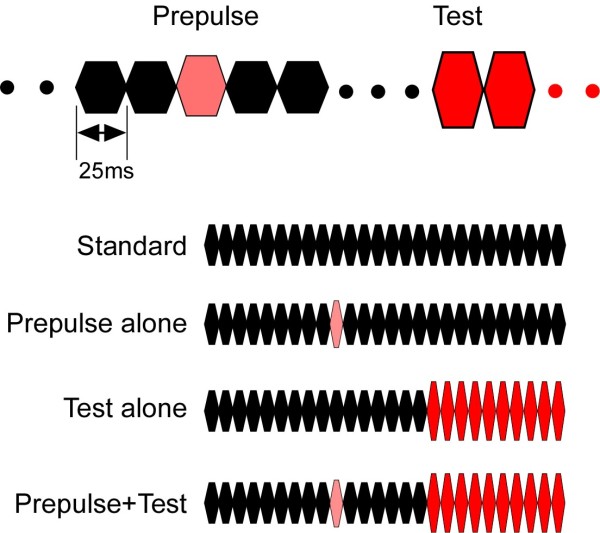
**Auditory stimuli.** The standard or background stimulus was a train of brief sounds 25 ms in duration, 800 Hz in frequency, and 65 dB SPL in sound pressure. The test stimulus to evoke change-related cortical responses was a similar train of 10 brief sounds of 70 dB. One brief sound was inserted before the test stimulus as a prepulse.

For auditory stimuli, we used a train of brief tone pulses [8,9]. The brief tone was 800 Hz in frequency, 25 ms in length (5 ms rise/fall), and 65 dB SPL in sound pressure. By using a train of brief standard tones followed by physically different tones, we could easily create an abruptly changing tone stimulus. When any changes occur in a continuous sound, a clear cortical response peaking at around 130 ms is recorded using EEG (Change-N1) or MEG (Change-N1m) [9,10,25]. In the present study, we used an abrupt increase of sound pressure by 5 dB to evoke Change-N1m, and effects of a preceding weak and brief change of sound pressure on Change-N1m were assessed by using a conditioning-test paired stimulation paradigm. In this study, we used four types of sound stimuli (Figure 1): 26 repeats of the same 65-dB brief tone 650 ms in total duration (Standard), 16 standard brief tones (400 ms) followed by 10 tones of 70 dB (Test alone), the Test preceded by one brief tone (prepulse) that was stronger than the standard tone but the same as or weaker than the Test (Prepulse+Test), and the Standard with a prepulse (Prepulse alone). Sound stimuli were presented binaurally through ear pieces (E-A-Rtone 3A, Aero Company, Indianapolis, IN).

### MEG recordings

Magnetic signals were recorded using a 306-channel whole-head type MEG system (Vector-view, ELEKTA Neuromag, Helsinki, Finland), which comprised 102 identical triple sensor elements. Each sensor element consisted of two orthogonal planar gradiometers and one magnetometer coupled to a multi-superconducting quantum interference device (SQUID) and thus provided 3 independent measurements of the magnetic fields. In this study, we analyzed MEG signals recorded from 204 planar-type gradiometers. These planar gradiometers are powerful enough to detect the largest signal just over local cerebral sources. The signals were recorded with a bandpass filter of 0.1-200 Hz and digitized at 1004 Hz. The analysis was conducted from 100 ms before to 700 ms after the onset of each stimulus. Epochs with MEG signals larger than 2.7 pT / cm were rejected from the averaging.

### Procedures

The experiments were conducted in a quiet, magnetically shielded room. The subjects sat in a chair and watched a silent movie on a screen 1.5 m in front of them throughout the experiments. Experiments 1 and 2 were carried out in this order on all subjects and separated by a 7 ~ 10-day interval. Two experiments were carried out at almost the same time of the day in each subject. An additional experiment was carried out on three subjects.

#### Experiment 1

To assess effects of the intensity of the prepulse, prepulses of 65.5, 66.5, 68, and 70 dB SPL (0.5, 1.5, 3, and 5 dB above the Standard, respectively) were used as the conditioning (prepulse) stimulus. Therefore, there were ten stimuli: 1) Standard alone, 2) Test alone, 3) ~ 6) Prepulse alone, and 7) ~ 10) Prepulse + Test. In the 3) ~ 10) stimuli, the tenth pulse (225 ~ 250 ms) was the prepulse. Since the Test started at 400 ms, the interstimulus interval (ISI) between the offset of the prepulse and the onset of the test was 150 ms. In this study, the prepulse-test interval was expressed as the ISI. Ten stimuli were presented randomly at an even probability at a trial-trial interval of 900 ms. For each stimulus, 150 ~ 155 artifact-free epochs were averaged.

#### Experiment 2

To assess effects of the interval between the prepulse and the Test, ISIs of 50, 100, 200, and 350 ms were used. The Test was the same as in Experiment 1. The prepulse was a 25-ms tone 67 dB in sound pressure (2 dB above the Standard). Similar to Experiment 1, there were ten stimuli that were presented randomly at an even probability at a trial-trial interval of 900 ms. For each stimulus, 150 ~ 155 artifact-free epochs were averaged.

#### Experiment 3

To examine whether a subtle change other than the sound pressure increase affects the Test-evoked cortical responses, two 25-ms prepulses of a decrease of sound pressure (3 dB below the Standard) and a frequency change (from 800 to 816 Hz) were used as the conditioning stimulus. There were six stimuli that were presented randomly: 1) Standard alone; 2) Test alone; 3) and 4) Prepulse alone (sound pressure decrease and frequency change); and 5) and 6) Prepulse + Test. The ISI between the prepulse and the test stimulus was 75 ms. The trial-trial interval was 900 ms. For each stimulus, 150 ~ 155 artifact-free epochs were averaged.

### Analyses

**Figure 2:**
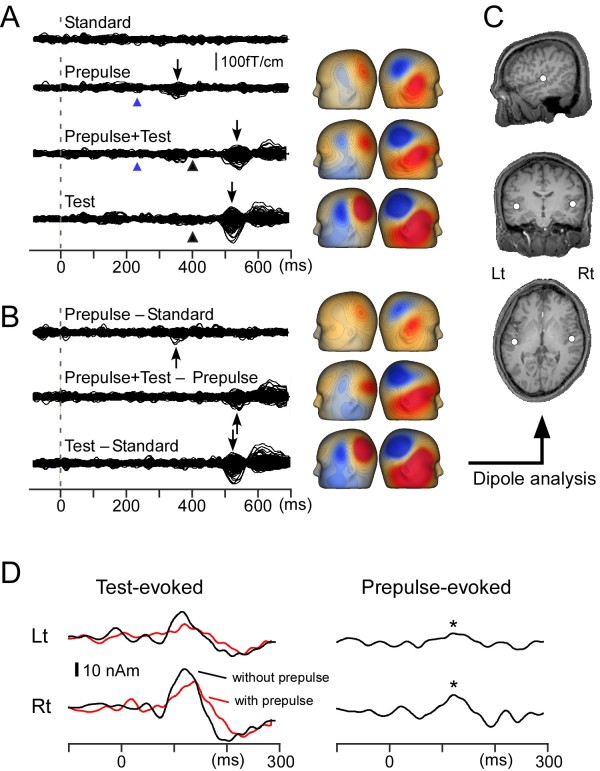
**Magnetic responses to the stimuli.** Data from one representative subject (Subject 4) for the Standard, 3-dB prepulse alone, Prepulse + Test and Test alone stimuli. **A**, super imposed waveforms of all the 204 sensors. Blue and black arrowheads indicate the onset of the prepulse and the test stimulus, respectively. Isocontour maps at the peak of the main magnetic component (Change-N1m) indicated by arrows are shown. **B**, superimposed difference waveforms and isocontour maps at the peak. **C**, location of the estimated dipole for Change-N1m superimposed on the subject’s own MR images. **D**, time course of the source strength waveform of the Test-evoked response (left) and Prepulse-evoked response (right). Black and red lines for the Test-evoked response indicate a source strength waveform for the test response following the Test-alone and Prepulse + Test stimulus, respectively. Based on the criteria used in this study, the Prepulse stimulus evoked a significant response (indicated by asterisks).

In Figure 2, procedures to analyze the evoked response are shown using data for a representative subject. Recorded MEG waveforms were subjected to band-pass filtering of 1 ~ 35 Hz [9,26]. To obtain Prepulse-evoked magnetic responses, a difference waveform was calculated by subtracting the waveform for the Standard from that for the Prepulse alone. The Test-evoked magnetic response was obtained by subtracting the waveform for the Prepulse alone from that for the Prepulse + Test stimulus (Figure 2B). The difference waveforms were used for analyses. An equivalent current dipole for the main component of the change-related responses, Change-N1m, was estimated for each hemisphere by use of BESA (NeuroScan, Mclean, VA) as described elsewhere [26,27]. Since the main purpose of the present study was to clarify whether the prepulse inhibits the test response, the dipole analysis was performed only for the Test-evoked magnetic response for the Test alone stimulus (Figure 2C), and the model obtained was applied to all the difference waveforms in the same experiment. Then the peak amplitude and latency were measured using the source strength waveform (Figure 2D). In the following text, the cortical response indicates the time course of the strength of a dipole (source strength waveform). The Test-evoked response indicates source strength waveform for the difference magnetic waveform obtained by subtracting the magnetic waveform for the Prepulse alone stimulus from that for the Prepulse + Test stimulus. The Prepulse-evoked response indicates the source strength waveform for the difference magnetic waveform obtained by subtracting the magnetic waveform for the Standard stimulus from that for the Prepulse alone stimulus (Figure 2D). The Test-evoked response for the Test alone stimulus was obtained by subtracting the waveform for the Standard from that for the Test alone stimulus. In the present study, the peak amplitude of Change-N1m was the amplitude between the peak of Change-N1m around 130 ms and the peak of a polarity-reversed earlier component around 60 ms [23,26]. This procedure minimizes problems due to a baseline shift. As compared to the response to the Test, the cortical response to the Prepulse is weak. We defined the Prepulse-evoked response as significant when the peak amplitude was larger than 3 SD of the prestimulus baseline. The percentage inhibition of the amplitude of the test response by the Prepulse (%PPI) was defined as (Test-evoked response to the Test alone stimulus – Test-evoked response to the Prepulse + Test stimulus) / Test-evoked response to the Test alone stimulus * 100.

The location of estimated dipoles was expressed in Talairach coordinates by using BESA and Brain Voyager (QX 1.4, Maastricht, The Netherlands). For both Experiment 1 and 2, statistical differences were evaluated using a one-way analysis of variance (ANOVA). When the sphericity assumption was violated, the Greenhouse-Geisser correction coefficient epsilon was used for correcting the degrees of freedom and then the F-value and significance probability were re-calculated. To compare the difference between conditions, post-hoc multiple comparisons were done with Bonferroni-adjusted t-tests. All statistical analyses were performed at the 0.05 level of significance. Data are expressed as the mean ± standard deviation (SD).

## Results

In all the 12 subjects, the Test, an abrupt increase of sound pressure by 5 dB, evoked a clear Change-N1m. The dipole for the Change-N1m was estimated to be located in the supratemporal plane around the superior temporal gyrus (STG) as described previously [8,10,11,23]. The mean x, y, and z Talairach coordinates of the dipole were −51, -23, and 5 for the left hemisphere, and 54, -17, and 4 for the right hemisphere in Experiment 1. Respective values of Experiment 2 were −53, -22, and 4 for the left hemisphere, and 54, -17, and 4 for the right.

### Experiment 1

**Figure 3:**
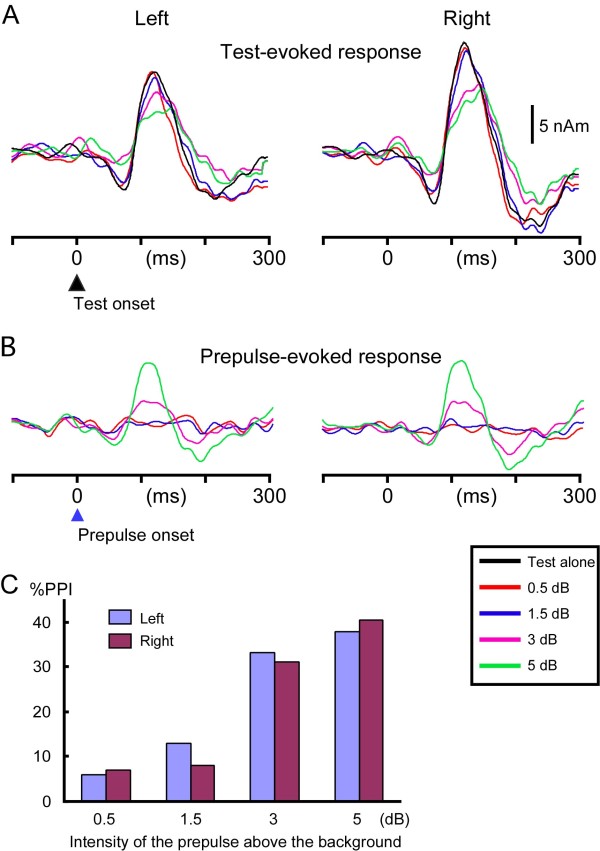
**Effects of the intensity of the prepulse on auditory Change-N1m.** Grand-averaged source strength waveforms of the Test-evoked **(A)** and Prepulse-evoked **(B)** response. **C**, the mean degree of the inhibition (percent prepulse inhibition, %PPI) for four prepulses.

The mean peak amplitude and latency of Test-evoked Change-N1m of the five conditions are listed in Table 1. For instructive purposes, grand-averaged waveforms across subjects are shown in Figure 3A. In both hemispheres, the Change-N1m amplitude decreased with the increase in the intensity of the prepulse (in dB above the background) in a linear fashion. The difference among conditions was significant (F (1.7, 19.0) = 11.4, p = 0.001, partial η^2^ = 0.51 for the left hemisphere; F (1.6, 18.1) = 13.2, p = 0.001, partial η^2^ = 0.55 for the right hemisphere). Post-hoc tests indicated that the Change-N1m amplitude of the 3-dB (p = 0.04) and 5-dB (p = 0.01) prepulse condition was significantly smaller than that of the Test-alone condition in the left hemisphere. Also in the right hemisphere, the amplitude of the 3-dB (p = 0.03) and 5-dB (p = 0.01) conditions was significantly smaller than the Test. The mean %PPI (Figure 3C) was 5.9 ± 14, 13.0 ± 21.7, 33.3 ± 17.8, and 38.0 ± 14.7% for 0.5, 1.5, 3, and 5 dB prepulses, respectively in the left hemisphere, and the difference among conditions was significant (F (3, 33) = 15.7, p < 0.001, partial η^2^ = 0.59). In the right hemisphere, the respective value was 7.0 ± 16.7, 8.0 ± 18.0, 31.0 ± 23.9, and 40.5 ± 14.7% (F (3, 33) = 18.2, p < 0.001, partial η^2^ = 0.62). The mean peak latency of Change N1m increased with the increase in the prepulse intensity (Table 1), but the difference was not significant (F (4, 44) = 1.0, p = 0.39 for the left hemisphere; F (4, 44) = 2.4, p = 0.063 for the right hemisphere).

**Table 1:** The peak latency and amplitude of Change-N1m

**Experiment 1**				
	**Peak Latency (ms)**		**Peak amplitude (nAm)**	
	**Lt**	**Rt**	**Lt**	**Rt**
Test	124±13	123±15	19.2±8.4	25.4±11.1
0.5dB	125±17	123±16	18.5±9.2	23.4±10.5
1.5dB	124±12	129±15	16.5±6.8	23.0±9.3
3dB	130±13	131±16	12.2±4.6	16.0±4.7
5dB	131±19	131±17	11.3±4.2	14.1±4.7
**Experiment 2**				
	**Peak Latency (ms)**		**Peak amplitude (nAm)**	
	**Lt**	**Rt**	**Lt**	**Rt**
Test	124±11	123±14	19.7±9.7	26.4±11.1
50 ms	131±13	134±19	12.5±4.9	15.9±5.7
100 ms	135±12	134±11	14.4±7.2	19.7±8.5
200 ms	126±12	128±13	16.9±10.6	24.4±8.7
350 ms	126±15	127±14	18.7±10.3	24.9±10.7

Values are the mean ± SD.

A significant response (> baseline + 3SD) to the prepulse itself (Figure 3B) was elicited by the 0.5, 1.5, 3, and 5-dB prepulse in 0, 1, 5, and 9 subjects, respectively in the left hemisphere, and 0, 1, 7, and 10 subjects in the right hemisphere. The amplitude of the Prepulse-evoked response in both the 3-dB and 5-dB prepulse conditions correlated linearly with the amplitude of the Test-evoked response for the Test-alone stimulus (r^2^ = 0.79 and 0.81).

### Experiment 2

**Figure 4:**
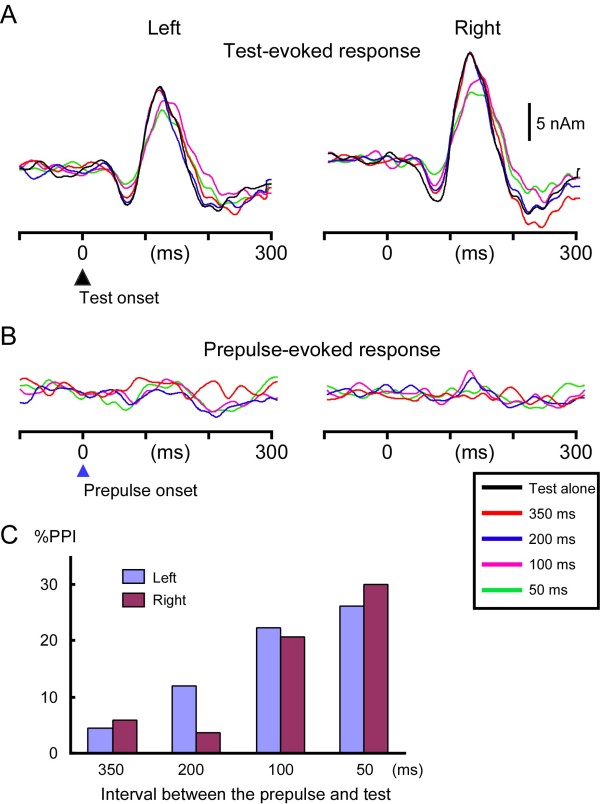
**Effects of the Prepulse-to-Test interval on auditory Change-N1m.** Grand-averaged source strength waveforms of the Test-evoked **(A)** and Prepulse-evoked **(B)** response. **C**, the mean %PPI for four interval conditions.

The mean amplitude and latency of the Test-evoked Change-N1m in all conditions are listed in Table 1. The grand-averaged waveform is shown in Figure 4. In both hemispheres, the Change-N1m amplitude decreased with the decrease in the ISI (F (4, 44) = 4.7, p =0.003, partial η^2^ = 0.30 for the left hemisphere; F (1.6, 18.1) = 10.0, p = 0.002, partial η^2^ = 0.48 for the right hemisphere). As compared to the Test alone condition, the Change-N1m amplitude was significantly smaller for the 100-ms ISI (p = 0.03) and 50-ms ISI (p = 0.03) conditions in the right hemisphere. The mean %PPI (Figure 4C) was 4.4 ± 21.8, 12.0 ± 24.6, 22.4 ± 24.8, and 26.1 ± 26.1% for the 350, 200, 100, and 50-ms ISI, respectively in the left hemisphere (F (3, 33) = 3.5, p = 0.025, partial η^2^ = 0.24). In the right hemisphere, the respective %PPI was 5.9 ± 13.0, 3.8 ± 10.3, 20.8 ± 17.1, and 30.0 ± 22.5 (F (1.6, 17.9) = 8.8, p = 0.003, partial η^2^ = 0.44). The peak latency of Test-evoked Change-N1m was longer for the shorter ISI prepulse condition (Table 1). The difference among conditions was significant (F (4, 44) = 4.9, p = 0.002, partial η^2^ = 0.31 for the left hemisphere; F (2.0, 22.3) = 4.9, p = 0.017, partial η^2^ = 0.31 for the right hemisphere). The prepulse elicited a significant Prepulse-evoked response in only 1 ~ 2 subjects in each ISI condition (Figure 4B).

### Experiment 3

**Figure 5:**
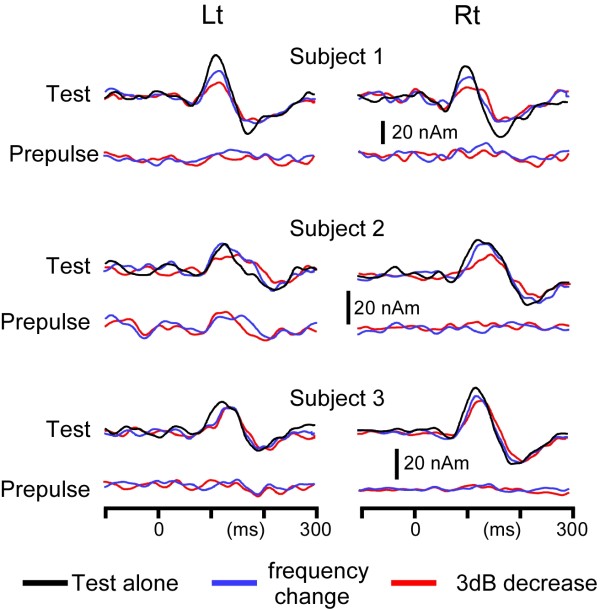
**Effects of prepulses of a subtle sound decrease and sound frequency change on auditory Change-N1m.** Test-evoked and Prepulse-evoked responses in all the three subjects (Subject 1 ~ 3) tested. The test response was elicited by an abrupt increase of sound pressure by 5 dB like in Experiment 1 and 2, while a brief sound with a weaker sound pressure (3 dB) or a higher sound frequency (2%) than the background was used as the prepulse. Note the very weak Prepulse-evoked response if present but clear reduction of the Test-evoked response.

Test- and Prepulse-evoked responses of the three subjects tested are shown in Figure 5. In all three subjects, the 3dB-down prepulse attenuated the Change-N1m amplitude (%PPI; 54, 39, and 6% for the left hemisphere and 51, 42, and 33% for the right hemisphere). The prepulse of a subtle frequency change attenuated Change-N1m clearly in the right hemisphere in all subjects (41, 18, and 15%), but for the left hemisphere, the inhibitory effect was weaker (35, -3, and 5%). Like in Experiment 1 and 2, the peak latency tended to be longer when the prepulse was present. The prepulse-evoked response was absent or very weak.

### Test-retest reliability, and correlation between the degree of PPI and change-N1m amplitude

**Figure 6:**
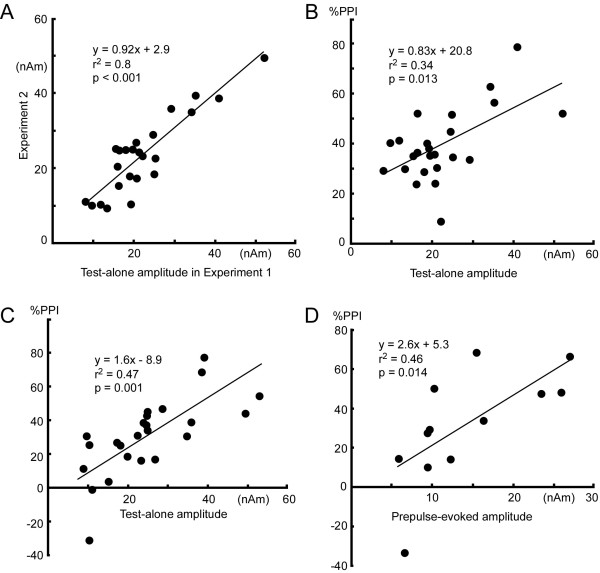
**Test-retest reliability (A) and correlation of the Change-N1m amplitude with %PPI (B~D).A**, plots of the amplitude of the Test-evoked response to the Test alone stimulus in Experiment 2 against that in Experiment 1 of both hemispheres of all the subjects. **B**, correlation between the amplitude of the Test-evoked response to the Test-alone stimulus and %PPI for the 3-dB prepulse condition in Experiment 1. **C**, correlation between the amplitude of the Test-evoked response to the Test-alone stimulus and %PPI for the 50-ms ISI condition in Experiment 2. **D**, correlation between the Prepulse-evoked amplitude and %PPI for the 3-dB condition in Experiment 1. All the results show that subjects with a greater Test-evoked response have a greater Prepulse-evoked response and are more strongly inhibited by the prepulse at least under these conditions.

The amplitude of the Test-evoked Change-N1m for the Test alone stimulus was compared between Experiment 1 and 2. Data plots in Figure 6A show the Change-N1m amplitude of Experiment 2 (y-axis) against that for Experiment 1 (x-axis) in both hemispheres of all the subjects. Both the slope of the regression line (0.92) and the coefficient (r^2^ = 0.8) show that Change-N1m is stable and therefore suitable for comparison among conditions confirming a recent study [26]. Scatter plots in Figure 6B show correlation between the amplitude of Change-N1m for the Test alone stimulus and %PPI by the 5-dB prepulse in Experiment 1. There was a linear correlation (p = 0.003). Results of similar analyses showed a significant correlation for the 3-dB prepulse (r^2^ = 0.25, p = 0.013) but not for the 1.5-dB (p = 0.65) and 0.5-dB (p = 0.7) prepulse. Similarly, there was a clear positive correlation between the amplitude of the Test-evoked response to the Test alone stimulus and %PPI for the prepulse of 50ms-ISI in Experiment 2 (p = 0.001, Figure 6C) but not other ISIs (P = 0.17 ~ 0.72).

Similar analyses were conducted for the relationship between the amplitude of the Prepulse-evoked response and %PPI in Experiment 1. Among the 12 hemispheres with a significant Prepulse-evoked response in the 3-dB condition, the %PPI correlated significantly with the amplitude of the Prepulse-evoked response (r^2^ = 0.46, p = 0.014)(Figure 6D). However, when %PPI was compared between the 12 hemispheres with a significant Prepulse-evoked response and 12 hemispheres without a significant response, %PPI was not different (31.3 ± 28 and 32.9 ± 10.3%). Among 19 hemispheres with a significant Prepulse-evoked response in the 5-dB prepulse condition, %PPI correlated significantly with the amplitude of the Prepulse-evoked response (r^2^ = 0.39, p = 0.004).

## Discussion

The present study demonstrated that auditory change-related cortical responses to an abrupt increase of sound pressure are attenuated by a preceding weaker and briefer change stimulus (prepulse) in a similar manner to the PPI of startle responses. That is, the degree of inhibition depends on the intensity of the prepulse and the time between the prepulse and test stimulus. The inhibition occurs even when the prepulse itself does not evoke a significant response, and the degree of inhibition appears to reflect the subject’s inherent sensitivity to a sensory change. Although further studies are necessary to determine whether the present phenomenon and PPI of the startle reflex have common physiological significance or mechanisms, prepulse inhibition of the cerebral response would advance our understanding of the mechanisms underlying sensory gating or its deficits in certain disease such as schizophrenia.

The findings in Experiment 1 that %PPI increases with an increase in the prepulse intensity is consistent with PPI of startle reflexes, in which stronger or more salient prepulses induce greater inhibition in general [28-31]. Similar to PPI of startle reflexes in humans [32] and mice [33], %PPI was positively correlated with the amplitude of the Prepulse-evoked response (Figure 6D). We consider that the prepulse and test stimulus activate a similar, or even identical, group of neurons reflected by Change-N1m, that greater Prepulse-evoked responses inhibit the test response more strongly, and, therefore, that subjects who are more sensitive to an abrupt sensory change are more strongly inhibited by the prepulse, which is in line with the protective hypothesis of PPI [1]. However, results of Experiment 1 in which %PPI was not influenced by the presence of the significant Prepulse-evoked response suggest that PPI can happen when the Prepulse-alone response is under the detection threshold as in PPI of the startle reflex [34], and that absence of the Prepulse-alone response in such a case does not mean an absence of brain responses to shape Change-N1m or to inhibit the Test response.

Results of Experiment 2 showed that %PPI depends on the interval between the Prepulse and Test similar to PPI of the startle response in which an ISI of 30 ~ 240 ms is used for eliciting PPI [2]. In the present study (Experiment 2), a shorter ISI produced greater PPI. However, as shown in Figure 6C, %PPI of the 50-ms ISI distributed widely from −31 to 77%. In 10 of 24 hemispheres, %PPI was smaller for the 50-ms than 100-ms ISI, which might be due to prepulse facilitation at a short ISI [2]. It seems possible in non-sensitive subjects that a prepulse at a short ISI serves as a part of the Test stimulus and enhances the response. From the viewpoint that PPI can be used as a powerful endophenotype in studies of schizophrenia [5], such a broad distribution may be of benefit.

Results of Experiment 3 demonstrated that the test response evoke by an abrupt increase of sound pressure can be inhibited by a prepulse with a change in different auditory features. We consider that this also supports the protective hypothesis since any detectable changes activate the change-detecting system, and once the system is activated, its activity should be protected for a certain period to complete the processing from being interfered with by succeeding events. The results may be useful to design the best protocol to use PPI of cortical responses, that is, Change-N1m can be elicited by any auditory change including frequency, intensity, and sound location [9], and a brief preceding sound with any feature change from the background can be a prepulse.

Like in our previous studies (e.g. [23]), the change-related response tended to be larger in amplitude in the right hemisphere (t(11) = 3.87, p = 0.003) for Experiment 1, t(11) = 2.13, p = 0.006 for Experiment 2) implying right hemisphere dominance for change detection. However, neither the main effect of the hemisphere nor the interaction with the experimental conditions was significant (ANOVA) for PPI. Since the present study used a fixed sound pressure level for both ears, a small difference in the hearing threshold between the ears might result in differences observed for the hemispheres. For evaluation of the hemispheric differences for the change-related response and its PPI, careful adjustment of the sound pressure level and detailed information of the handedness appear to be necessary.

## Conclusion

Here, we demonstrated that auditory change-related cortical responses are inhibited by a prepulse in a similar manner to PPI of startle reflexes. Since auditory Change-N1m is 1) easily recorded using EEG within a few minutes [9,12], 2) evoked by a small deviance of a sound feature without using an intense stimulus, and 3) stable like the blink reflex and its PPI are [35-37], it would be a valuable tool for understanding mechanisms of sensory gating or its deficits in a disease such as schizophrenia. In future studies, PPI should be compared directly between the startle reflexes and cortical responses to find similarities and dissimilarities. Comparisons with other experimental measures of inhibitory control, such as P50 gating [38] or PPI of perceived intensity [39], are also necessary. Another potential future study is a genetic [40,41] or pharmacological study. For example, nicotine is known to augment PPI of the startle reflex [42] as well as the generation of Change-N1m [26].

## Abbreviations

EEG: Electroencephalography; ISI: Interstimulus interval; MEG: Magnetoencephalography, PPI, Prepulse inhibition.

## Competing interests

The authors declare that they have no competing interests.

## Authors’ contributions

KI contributed to planning the study, data collection and analysis, and drafting the paper. EM, HT and MN contributed to planning the study. AT and MK contributed to data collection and analysis. SK and RK contributed to drafting the paper. All authors read and approved the final manuscript.
